# Harnessing evanescent photoacoustic waves for multi-domain imaging

**DOI:** 10.1016/j.pacs.2025.100719

**Published:** 2025-03-29

**Authors:** Rong Zhou, Liying Zhang, Beibei Li, Jingtao Xiao, Yiheng Xing, Chang Chen, Yuecheng Shen, Hao Shen, Deng Pan, Hongxing Xu

**Affiliations:** a State Key Laboratory of Precision Spectroscopy, East China Normal University, Shanghai 200241, China; b School of Physics and Technology, Wuhan University, Wuhan 430072, China

**Keywords:** Photoacoustic imaging, Acoustic evanescent wave, Supercritical angle emission

## Abstract

Photoacoustic microscopy (PAM) offers a non-invasive imaging method that overcomes the limitations of light scattering in biological tissues by visualizing optical contrast through the detection of photo-generated acoustic signals. While optical microscopy has significantly advanced through the exploration of optical evanescent waves, the potential of evanescent photoacoustic (PA) waves in PAM remains largely unexplored. In this work, we demonstrate the generation and detection of evanescent PA waves in PAM by positioning the sample near an interface, which directs these waves into the far-field beyond the supercritical angle (SA). These SA-PA signals exhibit distinct characteristics, including dependence of intensity on the source depths and symmetry in far-field angular patterns. Leveraging these features, we develop a proof-of-concept for supercritical angle photoacoustic microscopy (SA-PAM), which utilizes evanescent PA waves to enable new PAM functionalities, such as surface topography reconstruction and edge detection. This approach highlights the role of acoustic near-field exploration in advancing PA technology.

## 1 Introduction

When waves encounter an interface from a medium with a lower wave speed to one with a higher wave speed, total internal reflection (TIR) occurs if the incidence angle exceeds the critical angle. Despite complete reflection, TIR gives rise to an evanescent wave within the second medium, confined to the vicinity of the interface. This phenomenon, traditionally a focus in optics, has been instrumental in the development of near-field optics [1], [2]. Within the realm of optical microscopy, the evanescent waves associated with TIR illumination can selectively excite fluorophores only near the interface [3], [4], [5].

Although TIR of incident propagating waves confines transmitted fields to the interface, evanescent incident fields can enable transmission beyond the critical angle. For instance, optical point sources, such as fluorescent molecules deposited on a glass substrate, generate near-fields that leak into the substrate beyond the critical angle [1], [6], [7], [8], [9]. This “supercritical angle (SA) emission” is a cornerstone of near-field optics and underpins supercritical angle fluorescence (SAF) microscopy [10], [11], [12]. SAF microscopy leverages the high localization of evanescent fields to achieve nanometric precision in axial positioning [13], [14], [15], [16], [17], [18], [19], making it an important compensation for super-resolution techniques like single-molecule localization microscopy [20], [21], [22], [23].

While optical microscopy is widely used, it faces limitations in scattering samples, such as biological tissues. Photoacoustic microscopy (PAM) [24], [25] overcomes this challenge by exploiting weak acoustic scattering, visualizing optical absorption contrast through the photoacoustic (PA) effect—where pulsed laser irradiation generates ultrasonic waves via thermoelastic expansion. Recent advancements in PAM have focused on manipulating the incident light field to improve resolution or light propagation in scattering media, using techniques like diffraction-limited focused beams [26], [27], optical fringes [28], wave-front pre-shaping [29], [30], [31], diffractionless beams [32], [33], [34] or employing temporal modulations like dual-pulse excitation [35]. Additionally, optical evanescent waves have been leveraged in PAM to enhance sensitivity or spatial resolution [36], [37], [38].

Despite the interplay of optical and acoustic fields in PAM, the acoustic field has primarily been utilized for its time-of-flight information [39] detected along a single specified direction. However, the potential of acoustic evanescent waves generated during the PA process and their implications for PAM has been largely overlooked. Similar to the role of optical evanescent waves in SAF microscopy, these evanescent PA waves could be decoded from the far-field angular distribution. They may encode rich information about subsurface structures, offering a new dimension for contrast and resolution in PAM. By combining these capabilities with the inherent advantages of PAM, this approach could lead to a powerful composite imaging method, advancing applications in biomedical imaging and beyond.

In this work, we uncover the intriguing phenomenon of evanescent PA waves associated with the PA process. These evanescent PA waves are universally generated when the laser is tightly focused below the acoustic wavelength of interest. Such evanescent PA waves can be directed into the far-field by adjacent local structures; here, we use an interface as an example, which redirects the evanescent PA waves into the far-field beyond the critical angle. These signals exhibit unique characteristics, such as depth-dependent intensity and symmetry in far-field angular patterns. To demonstrate the potential applications of evanescent PA waves in photoacoustic microscopy (PAM), we introduce a proof-of-concept for supercritical angle photoacoustic microscopy (SA-PAM). This technique utilizes evanescent PA waves to achieve advanced functionalities, including surface topography reconstruction and edge detection. Consequently, our work not only reveals the presence and properties of evanescent PA waves in PAM but also highlights their potential for advancing PA technology.

## 2 Evanescent PA waves

Evanescent photoacoustic (PA) waves are a ubiquitous phenomenon in PA processes when the incident laser is tightly focused to a spot size smaller than the acoustic wavelength. Under such tight focusing conditions, the absorption of laser irradiation induces rapid thermal expansion confined to the laser focal area. When the laser focus is smaller than the acoustic wavelength, the confined expansion profile contains Fourier components with imaginary wave vectors, leading to the generation of evanescent PA waves.

**Fig. 1:**
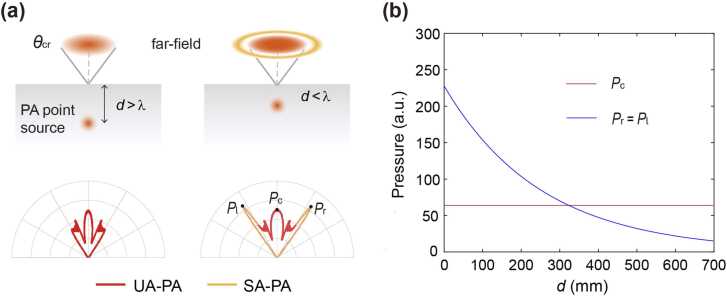
Revealing PA evanescent waves near an interface. (a) Top: focused lasers excite PA point sources (red points) on the sample surface at depths d from the interface. Bottom: numerical results of corresponding far-field PA signals. When $d>\lambda_{S}$ (S-wave wavelength in glass), PA signals are confined within the critical angle (UA-PA signals, red curves). For $d<\lambda_{S}$, in addition to UA-PA signals, evanescent waves associated with the PA point source leak into the SA region (SA-PA signals, orange curves), resulting in a three-lobe far-field pattern with peak values denoted as $P_{l}$, $P_{r}$ and $P_{c}$. (b) Amplitude maxima in the far-field for different source depths.

To direct the evanescent PA waves into the far field, a local structure near the excitation position is required. In this study, we consider a solid-water interface with the sample embedded in the solid and deposited near the interface. The PA point source emission of such a sample is modeled by modeling an acoustic point source near the interface, as illustrated in Fig. 1(a). While the emission of an electromagnetic point source near an interface is a well-established concept in near-field optics [1], [6], [7], [8], [9], its acoustic counterpart has not yet been clearly addressed.

Compared to the optical counterpart, the acoustic interface considered here entails additional complexities, as solid materials like glass exhibit both S- and P-waves. Critical angles for both S- and P-waves can be determined by $\theta_{\mathrm{cr}}^{S,P}=\mathrm{asin}(c_{w}/c_{S,P})$, where $c_{w}$ and $c_{S,P}$ denote the sound speed in water (longitudinal wave) and solid (taking glass as an example), respectively. The velocities adhere to the relation $c_{P}>c_{S}>c_{w}$, resulting in $\theta_{\mathrm{cr}}^{S}>\theta_{\mathrm{cr}}^{P}$. This study exploits the SA-PA signals dominated by the transmission of the S-wave, measured at angles $\theta >\theta_{\mathrm{cr}}^{S}$. The contribution of the P-wave is minimal, as it decays faster than the S-wave along the z-axis. Consequently, unless specified otherwise, references to the critical angle and SA-PA signals pertain to the S-wave.

The emergence of SA-PA signals as a PA point source approaches the interface is numerically demonstrated as shown in Fig. 1(a). While the experimental PA signals span a frequency range, the physics can be captured by simulating a monochromatic acoustic point source, aligning its frequency (f=5 MHz) with the central of the PA spectrum (see Supplementary Note). Additionally, we model the point source with an initial velocity perpendicular to the interface to capture thermal expansion details (see Methods and Supplementary Note). For a large source depth $d=600\;\mu m>\lambda_{S}$ (left panel, Fig. 1(a)), where $\lambda_{S}=591\;\mu m$ is the S-wave wavelength in glass, PA signals resulting from the regular transmission of acoustic propagative waves are confined within the UA region (UA-PA signals). For a small depth $d=200\;\mu m<\lambda_{S}$ (right panel, Fig. 1(a)), additional distinct side lobes emerge in the angular distribution beyond the critical angle $\theta_{\mathrm{cr}}^{S}$. Notably, minima are observed at the critical angle $\theta_{\mathrm{cr}}^{S}$, contrasting with optical results [1], [6], [7], [8], [9], where maxima are observed along the critical angle. Experimental findings discussed below further corroborate these angular patterns. For clarity in further discussions, the maxima of the UA-PA central lobe and the two SA-PA lobes are denoted as $P_{c}$, $P_{l}$, and $P_{r}$, respectively (bottom right, Fig. 1(a)).

Given the complexities inherent in the acoustic scenario, the point-source-near-interface system exhibits intricate features in the far-field distribution. For instance, two minor side lobes emerge at angles $\theta_{\mathrm{cr}}^{S}>\theta >\theta_{\mathrm{cr}}^{P}$, accounting for the SA-PA signals of P-wave transmission (see Supplementary Note and experiment results presented below). Furthermore, the three lobe maxima that we focus on, denoted as $P_{c}$, $P_{l}$, and $P_{r}$, receive contributions of both P- and S-wave transmissions. Specifically, the central lobe peak at θ=0, i.e., $P_{c}$, originates solely from the P-wave associated with the point source, as symmetry requirements preclude the contribution of the S-wave. In contrast, the two side lobe maxima, $P_{l}$, and $P_{r}$, originate predominantly from the S-wave, given their distance from $\theta_{\mathrm{cr}}^{P}$, with minimal contribution of the P-wave (see Supplementary Note).

Despite the intricate differences and complexities in the acoustic case, the relationship between the far-field acoustic amplitude and the depth parameter d exhibits similarities with its optical counterpart. More precisely, the UA-PA signals remain largely insensitive to the distance d (Fig. 1(b)). In particular, the amplitude $P_{c}$ along the direction θ=0 theoretically maintains a constant value, determined by the normal transmission coefficient of P-wave. In contrast, the SA-PA signals, largely characterized by the values of $P_{l}$, and $P_{r}$, stem from the leakage of acoustic evanescent waves, displaying an exponential increase as the point source approaches in the interface (Fig. 1(b)).

Due to the translational symmetry of the interface, the signal observed at a specific direction θ results from the transmission of components, with the in-plane wave vector $q_{w}\sin(\theta )$ being conserved. The corresponding wave components in the glass acquire a *z*-component wave vector, $\kappa_{j}=\sqrt{q_{j}^{2}-q_{w}^{2}\sin^{2}(\theta )}$, where $q_{w}=2\pi f/c_{w}$ and $q_{j}=2\pi f/c_{j}\;(j=S,P)$ represent the wave vectors in water and glass, respectively. In the SA scenario where $\theta >\theta_{\mathrm{cr}}^{S}$, $\kappa_{j}$ becomes imaginary, indicating an evanescent component in the glass that decays exponentially. The transmitted amplitude of this evanescent component is characterized by the factor $e^{-|\kappa_{S}\mathrm{|d}}$ (as the contribution of the P-wave is minimal). Taking into account the above considerations, a dimensionless factor can be defined by $\eta =(P_{l}+P_{r})/2P_{c}$, satisfying the relation $\eta =\eta_{0}e^{-|\kappa_{S}\mathrm{|d}}$.

## 3 SA-PAM for multidomain imaging

### 3.1 Overview of SA-PAM

**Fig. 2:**
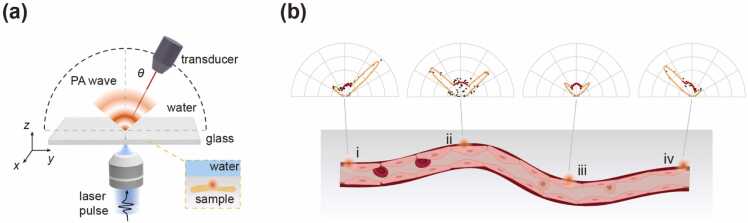
The concept of SA-PAM. (a) In angle-resolved PAM, a pulsed laser beam is focused onto the sample to generate PA signals. Their far-field angular distributions are acquired by a transducer rotating around the sample, while remaining confocal with the laser. In SA-PAM, the angle-resolved PAM is combined with a sample embedded near an interface (inset). (b) Multi-domain imaging capabilities of SA-PAM: the sensitivity of $\eta =(P_{l}+P_{r})/{2P}_{c}$ to d and the pattern asymmetry (characterized by $|P_{l}{-P}_{r}|$) at discontinuities are utilized for retrieving surface topography and edge information, respectively.

The setup and working principle of SA-PAM are illustrated in Fig. 2. SA-PAM is performed using a home-built angle-resolved photoacoustic microscopy (see Methods section and Supplementary Note for details). In angle-resolved PAM, a transducer detector collects the angular distribution of the PA signals by scanning around the sample while maintaining alignment (Fig. 2(a)). We note that the transducer rotation is solely for measuring 180° far-field angular patterns. For the other results presented below, including surface reconstruction and boundary detection, we employ a second scheme utilizing three fixed transducers that simultaneously record signals $P_{l}$, $P_{r}$ and $P_{c}$. This approach eliminates the need for transducer rotation.

All measurements are performed in a water environment. Similar to SAF microscopy, the sample in SA-PAM is embedded in a medium (inset, Fig. 2(a)) with higher sound speed (e.g., glass) and positioned close to its interface with water. Laser irradiation generates a point-like acoustic source at the focal point (red points, Fig. 2(a)). This photo-generated acoustic source, a PA point source, involves both propagating and evanescent acoustic wave components, leading to the generation of UA- and SA-PA signals, respectively.

The rich information encoded in the angular distribution of PA signals in SA-PAM opens exciting avenues to advance PAM beyond its conventional capabilities, as schematically illustrated in Fig. 2(b). Firstly, SA-PAM can be utilized for reconstructing sample surfaces by exploiting the one-to-one correspondence between the factor η and the depth parameter d, similar to the axial localization in SAF microscopy [13], [14], [15], [16], [17], [18], [19]. This allows for inferring the sample surface geometry, characterized by the depth parameter d(x,y) since a planar interface is used, from the spatial information of η(x,y) measured in SA-PAM (Fig. 2(b), panels ii and iii). This correspondence is precise for locations within the central area of the sample, where the angular pattern maintains good symmetry.

Secondly, SA-PAM proves effective in detecting structural discontinuities in samples, on such edges and boundaries between different tissues, through analysis of the far-field angular pattern. This parallels recent advancements in optical edge detection [40], [41], [42]. As illustrated in Fig. 2(b) (panels i and iv), laser focusing near the sample edge induces asymmetry in the far-field SA-PAM patterns, with emissions directed to the closer side lobe. This asymmetry arises from the interference between direct PA emissions and those re-scattered by the edge. The spatial information of SA-PAM asymmetry represented by $|P_{l}{-P}_{r}|$, signifies the contrast between the two sides of sample boundaries. It should be noted that the edge detection is generally achievable with angle-resolved PAM, and the presence of an interface in the sample is not a prerequisite. However, the significantly enhanced side lobe observed in SA-PAM confines the PA signals to a narrow angular region, thereby improving the signal-to-noise ratio.

### 3.2 Depth sensitivity and surface reconstruction in SA-PAM

**Fig. 3:**
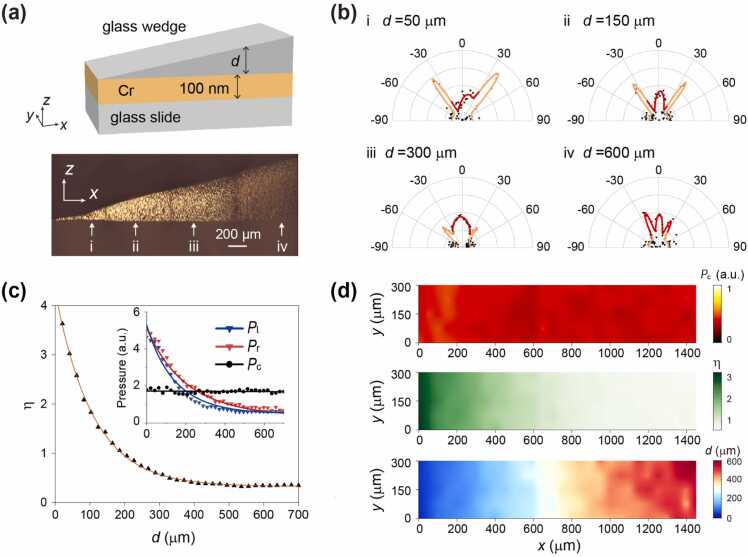
Depth resolution characterization in SA-PAM. (a) Thin film sample (Cr) embedded in glass with a continuously varying depth d from the upper interface. Upper: 3D schematics. Bottom: optical section view. (b) SA-PAM measurements for lasers tightly focused at four locations (i, ii, iii, and iv are indicated in (a), respectively), generating acoustic point sources with varying depths d. (c) Dependence of peak values ($P_{l}$, $P_{r}$ and $P_{c}$, inset) and the corresponding value of η on the depth d of the photo-generated acoustic point source. (d) 2D images of the sample using the center peak value $P_{c}$ (upper), the ratio η (middle), and the depth d, as indicated by the colored scale. The image of the depth d is retrieved from that of η, using their correspondence shown in (c).

To experimentally confirm the depth sensitivity of SA-PAM concerning the point sources generated near interfaces and its potential to reveal surface topography, a sample with a continuously variable depth parameter is designed (Fig. 3(a)). This sample employs a thin Cr film deposited on a glass substrate as the target, generating PA signals upon laser illumination. The film is covered by a wedge-shaped glass (see Methods and Supplementary Note), offering a continuous change in thickness of up to ∼700 μm. Focusing a laser onto the Cr film generated an acoustic point source, with its depth corresponding to the wedge thickness at the focal spot.

Characterization of the prepared sample using SA-PAM with laser beams focused at four distinct locations with varying depths is shown in Fig. 3(b). Consistent with both theoretical predictions and the optical counterpart, the SA-PA signals exhibit sensitivity to changes in the depth, whereas a rapid decline in the SA-PA signals is observed with increasing depth. Interestingly, at a large depth of d= 600 μm (panel iv, Fig. 3(b)), two minor lobes emerge, in consistence with the simulation result shown in Fig. 1(a), attributed to SA-PA signals from the P-wave in glass. The depth-dependent behavior of the UA- and SA-PA signals is further demonstrated by the variation of their peak values (inset, Fig. 3(c)). As d increases, $P_{c}$ remains constant, while $P_{l}$ and $P_{r}$ experience an exponential decrease. The derived dimensionless factor $\eta =(P_{l}+P_{r})/{2P}_{c}$, shown in Fig. 3(c), can be well-fitted by an exponential function, $\eta =\eta_{0}\exp\left(-\kappa d\right)+b$, yielding $\eta_{0}=3.93$, $\kappa =5.2\;{\mathrm{mm}}^{-1}$, and b=0.26.

The nonzero background value, b, represents noise originating from PA signals scattered at the boundaries of the finite-sized sample. This is further confirmed by numerical results (see Supplementary Note), showing a similar exponential decay of η with increasing d with a zero background (b=0). Proper sample design could further mitigate this noise. Furthermore, according to the proposed theory, the decaying parameter κ can be estimated using |$\kappa_{S}|$, where $\kappa_{S}=\sqrt{{q_{S}^{2}-q}_{w}^{2}\sin^{2}(\theta_{\mathrm{side}})}$, with wave vectors $q_{w}$ and $q_{S}$ calculated for the central frequency of 5 MHz. In our experimental setup, the transducer remains fixed at $\theta_{\mathrm{side}}=\pm 34{}^{\circ}$ to measure $P_{l}$ and $P_{r}$. With the sound speed in water ($c_{w}=1480\;m/s$) and the S-wave speed in glass ($c_{S}=2958\;m/s$) taken into account, we determine $\left|\kappa_{S}\right|$ to be $5.3\;{\mathrm{mm}}^{-1}$, demonstrating excellent agreement with the experimental findings.

The precise depth-dependence of the characteristic factor η enables the use of SA-PAM measurements to reconstruct the surface geometry of the sample. Fig. 3(d) presents 2D images of the sample using various quantities measured in SA-PAM. The image of $P_{c}$ (upper panel, Fig. 3(d)) corresponds to the conventional PAM result, yielding only a homogeneous image of the sample. In contrast, the images of $P_{r}$ and $P_{l}$, or equivalently, the factor η (middle panel, Fig. 3d), are encoded with the information of the sample surface. Employing the fitted relationship between η and d established in Fig. 3(c), the sample surface is reconstructed (bottom panel, Fig. 3(d)). The film surface appears as a slope relative to the upper interface, in consistence with the wedge shape of the cover glass.

### 3.3 Edge detection

**Fig. 4:**
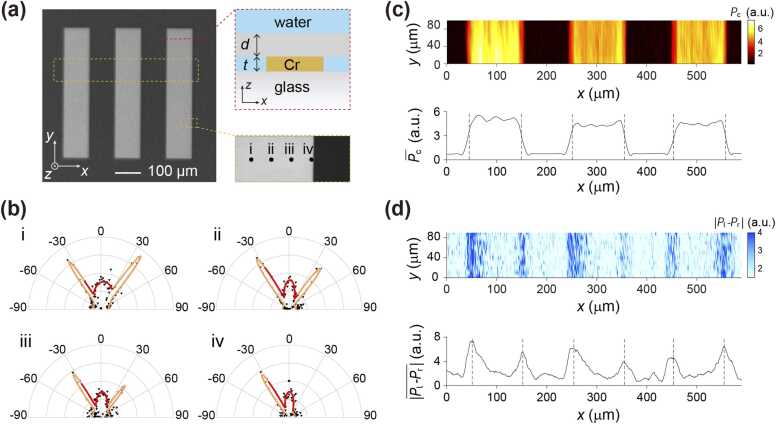
Edge detection in SA-PAM. (a) Optical images and schematic section view of the sample adopted for edge detection. The sample comprises a USAF-1951 target (*t* = 100 nm) covered with a *d*= 100 μm thick glass. (b) Angular far-field patterns acquired in SA-PAM for lasers focused at four locations near a sample edge (indicated in the bottom-right panel in (a). (c) Upper: conventional PAM image of the sample (upper dashed-rectangle area indicated in a) using the central peak value $P_{c}$. Bottom: average results of the image along the *y*-direction. (d) Same as (c), but plotted for the difference between side peak values, i.e., ${\mathrm{|P}}_{r}-P_{l}|$.

When the laser focus is distant from the sample edge (Fig. 3), far-field patterns consistently exhibit symmetry. Boundaries are always present in sample, as exemplified by a sample shown in Fig. 4(a), which comprises a USAF-1951 target covered by a 100 μm thick glass. As the laser focus approaches the edge of the Cr bar (see Fig. 4(a) and (b)), the asymmetry of the SA-PAM patterns becomes pronounced. More precisely, a linear attenuation of intensity is observed in the side lobe oriented outward the Cr region, while the intensity of the inward-oriented side lobe remains constant.

In a conventional PAM image of the sample, plotted using the central peak value $P_{c}$ (upper panel, Fig. 4(c)), uniform PA signal distributions are observed within the central area of Cr bars, decreasing only near the edges. Edge recognition in this image relies on the contrast between neighboring regions. In contrast, SA-PAM offers a more distinct approach to revealing edge information by exploiting the asymmetry of the SA-PAM pattern near the edge. This symmetry, characterized by $\left|P_{r}-P_{l}\right|$ and imaged in Fig. 4(d) (upper panel), exhibits maxima that delineate the contour of the Cr bars. The precision of this method is further confirmed by averaging the value of $\left|P_{r}-P_{l}\right|$ along the *y*-direction (lower panel, Fig. 4(d)), demonstrating a peak width of approximately 33 μm and a sharply defined tip. Comparison of these peak locations with the conventional PAM image (upper panel, Fig. 4(c)) and the *y*-direction average of the conventional PAM image (lower panel, Fig. 4(c)) demonstrates that $\left|P_{r}-P_{l}\right|$ measured in SA-PAM precisely reveals the edge locations.

### 3.4 Imaging honey bee wings by SA-PAM

**Fig. 5:**
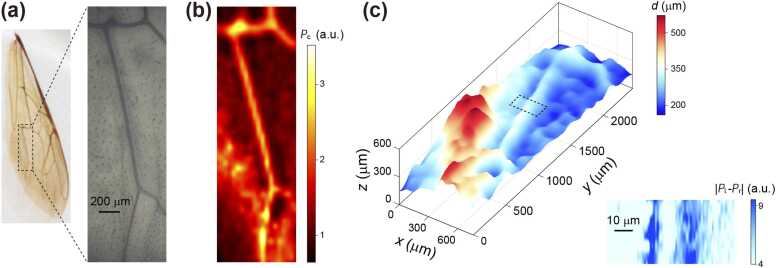
Characterizations of a bee wing using SA-PAM. (a) Optical image of a bee wing embedded in epoxy resin near a planar interface. (b) Conventional PAM image using the signal $P_{c}$. (c) Surface topography of the bee wing reconstructed using SA-PAM. Inset: imaging of the two boundaries of a vein (dashed square in the main panel) using SA-PAM.

To further demonstrate the capabilities of SA-PAM, we apply it to a biological sample: a honey bee (subspecies: Apis cerana) wing. This choice is motivated by the intricate structure of bee wings, offering an ideal test bed for our methodology. As required for SA-PAM characterization, the bee wing is embedded in epoxy resin and positioned within the acoustic wavelength scale near a planar upper interface. The optical photograph (Fig. 5(a)) and the conventional photoacoustic (PA) image (Fig. 5(b)) of the bee wing highlight the veins of the underwing. The color scale in the conventional PA image reveals information about the material composition, with stronger PA signals corresponding to the veins.

Characterization of the sample using SA-PAM is presented in Fig. 5(c), which reveals a precise reconstruction of the bee wing's topography (main panel). To accomplish this reconstruction, given the different materials used to embed the sample different glass, we follow the procedure in Fig. 3(c), recalibrate the parameters in the relation, $\eta =\eta_{0}\exp\left(-\kappa d\right)+b$, yielding $\eta_{0}=2.16$, κ=3.74 mm^−1^, and b=0.28 (see Supplementary Note). Through the same scanning measurement to acquire the topography Fig. 5(c), precise edge detection is simultaneously achieved (inset in Fig. 5(c)), where the bright signals distinctly reveal the two edges of a vein in the wing. The exquisite detail captured in the reconstructed topography underscores the system's good localization precision, evident in its ability to precisely delineate the subtle fluctuations of the wing veins and surface features.

## 4 Methods

### 4.1 SA-PAM setup

The SA-PAM setup is illustrated in Fig. 2(a), with a more detailed layout provided in Supplementary Note. The system employs a 532 nm pulsed laser (Surelite, Continuum) with a 1 ns pulse width and a 10 Hz repetition rate as the excitation source. To compensate for acoustic impedance mismatch at water-glass interfaces that induces approximately 70 % signal attenuation, the laser pulse energy was optimized to 0.8 μJ for Cr films and 2.3 μJ for bee wing specimens through a rigorous evaluation balancing signal-to-noise ratio and damage threshold. An objective (N.A.=0.3; Olympus) focused the laser onto the water-immersed sample, creating a diffraction-limited laser spot of ∼2 μm in diameter. Considering the potential photothermal damage, strategic defocusing was employed to form a laser illumination of 15 μm in diameter at the sample plane. Compared to the ∼300 μm wavelength of the studied 5 MHz PA signals in water (significantly longer in glass), the photo-generated acoustic source can be reasonably approximated as a point source. The sample was mounted on a motorized stage (MS-2000, ASI) and scanned in the *x*-*y* plane to acquire 2D images.

Generated PA signals were detected by a focused ultrasonic transducer (5 MHz center frequency; V309-SU, Olympus) immersed in water and aligned confocal with the laser. The detected signals were amplified by an electric amplifier (50 dB; ZFL-500LN, Mini-Circuits) and subsequently digitized and averaged by an oscilloscope (200 MHz bandwidth; MOS-X 3024 A, Agilent Technologies).

To capture the angular distribution of the PA signals, a transducer was mounted on a rotating platform controlled by a motorized system, comprising three-step motors and a motion control program. Two-step motors precisely positioned the transducer in the two orthogonal directions within the *x*-*z* plane. The transducer remains oriented within the *x*-*z* plane, and a third-step motor controlled the transducer’s rotation around its center. The program guided the transducer along a circular orbit with a fixed distance from the sample, while sweeping the azimuthal angle *θ*. During this process, the transducer remains aligned with laser focus by adaptively rotating the third step motor. A full 180° far-field angular scanning with an angular resolution of 3° per step requires an acquisition time ranging from 3 to 5 minutes. The final angular distribution pattern was derived through averaging multiple independent scans.

To enhance temporal resolution, we implemented an alternative acquisition scheme employing a fixed array of three transducers positioned at special angles. This configuration enables simultaneous recording of acoustic signals from multiple orientations (denoted as $P_{l}$, $P_{r}$ and $P_{c}$), effectively eliminating the need of mechanical rotation during the two-dimensional imaging process. The only instance where an angular sweep is required is the initial one-time scanning to determine critical angles and the angles of side lobes.

### 4.2 Numerical simulations

The finite element method (COMSOL Multiphysics™ 6.0, pressure acoustic module) was used to obtain the numerical simulation results presented in Fig. 1(a) (see more details in Supplementary Note 3). We modeled an acoustic point source positioned within a glass region, close to its interface with a water region. To prevent reflections, the simulation domain was enclosed by a perfectly matched layer (PML). The material properties were specified as follows: mass densities of 1000 kg/m³ for water and 2200 kg/m³ for glass, sound speed of $c_{w}=1480$ m/s in water, and P- and S-wave speeds of $c_{p}=5710$ m/s and $c_{s}=2958$ m/s in glass, respectively.

For solids like glass, the point source is modeled as a point with a unit velocity along a chosen direction. In Fig. 1(a), we assume that the point velocity is perpendicular to the interface (*z*-direction), reflecting the rotational symmetry of experimental configurations when the laser focus is far from sample boundaries. This symmetry permits capturing the essential physics using a 2D model coupled with a rotational axis. After calculating the field within the simulation domain, the far-field angular distribution was obtained by evaluating the Helmholtz-Kirchhoff integral.

### 4.3 Sample preparation

For the sample used in Fig. 3, a 100-μm chromium film was deposited on a glass substrate and then covered by a wedge-shaped cover glass. The wedge cover glass was fabricated using wet etching with a 5–10 % aqueous solution of hydrofluoric acid (CAS: 7664–39–3, Aladdin). To fabricate the wedge, a cover glass of uniform thickness was initially submerged vertically in the solution, with one side masked to prevent etching. The glass was then gradually withdrawn from the solution at a constant speed. This resulted in a longer etching time for the bottom portion of the glass compared to the top, leading to a wedge shape (see details in Supplementary Note). The angle of the wedge can be controlled by adjusting the pulling speed.

For the sample in Fig. 5, a honey bee wing was embedded in a solid block of epoxy resin for SA-PAM characterization. This preparation involved first mixing liquid epoxy resin with curing agent in a 3:1 ratio. We then poured the mixture into a 1-mm-thick square mold and carefully placed the bee wing within the liquid, ensuring it was close to the upper surface but tilted at an angle. This intricate 3D arrangement, involving both tissue structures and the overall surface depth distribution, was captured in the topography image shown in Fig. 5(c). To prevent bubble formation, the entire process was conducted in a vacuum environment. After approximately 36 hours, the liquid solidified into transparent, solid resin block with the embedded bee wing.

## 5 Discussion

### 5.1 Future refinement of SA-PAM

In this work, we introduce a proof-of-concept for SA-PAM to demonstrate the potential applications of evanescent PA waves in PAM. SA-PAM is specifically designed to characterize structures near interfaces and boundaries (within the acoustic wavelength range), achieving functionalities such as surface topography reconstruction and edge detection, which complement conventional PAM. By focusing on surface and boundary features, SA-PAM readily inherits the broad advantages of conventional PAM and can be applied to a wide range of scenarios. For instance, it can detect endogenous chromophores without fluorescent labels, enabling functional and molecular imaging near interfaces. It also provides critical functional data, such as blood oxygenation, metabolic activity, and microvascular dynamics near interfaces—capabilities that are challenging to achieve with standard optical microscopy. Unlike fluorescence microscopy, which risks photobleaching and phototoxicity, SA-PAM uses pulsed laser excitation and detects ultrasonic signals, minimizing photodamage and making it ideal for long-term live-cell and in vivo studies. Additionally, SA-PAM offers high optical absorption contrast, excelling in imaging vascular structures, tumors, and oxygen saturation levels near interfaces.

**Fig. 6:**
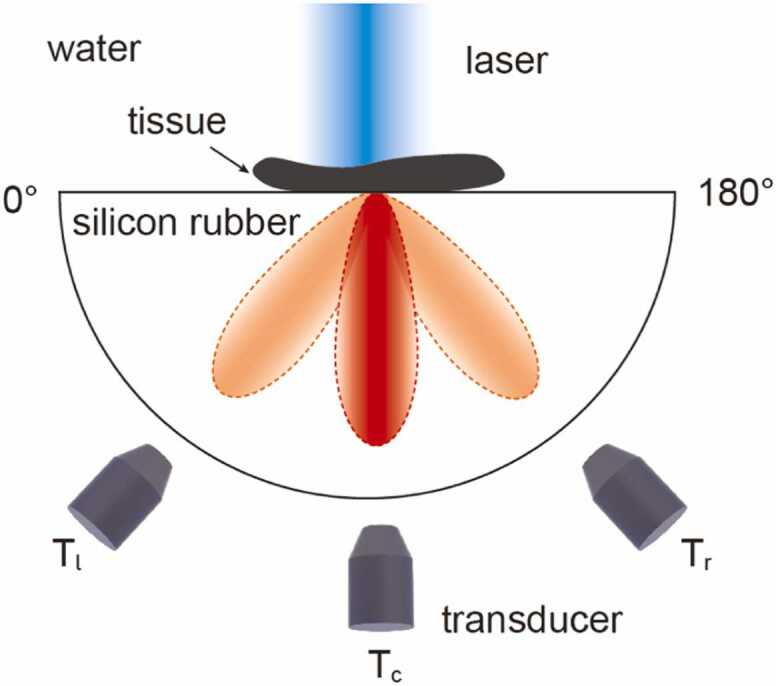
Schematic of improved SA-PAM experimental setup.

Since SA-PAM requires the presence of an interface in the sample, the experiments described in this work use samples sandwiched between two glass pieces or embedded in epoxy resin to provide such an interface. While this sample requirement may initially seem restrictive, it can be adapted to leverage naturally occurring or engineered interfaces, significantly broadening its applicability beyond the specific configurations demonstrated in our study. For example, we propose an alternative configuration, as illustrated in Fig. 6, where biological tissue is placed on a semi-hemispherical silicone rubber substrate. The speed of sound in water (1480 m/s) is close to that of biological tissues, while the sound speed in silicone rubber is approximately 1000 m/s [43]. The interface between water and silicone rubber supports the existence of a supercritical S-wave within the silicone rubber at an angle of around 42 degrees. Three transducers are positioned on the silicone rubber at angles of 48°, 132°, and 90° to separately collect the SA-PA and UA-PA signals. The far-field acoustic waves are transmitted normally to the cylindrical boundary of the silicone rubber and are captured by the three transducers. Additionally, we also note that interfaces are ubiquitous in both biological and industrial imaging applications, including even internal interfaces within biological tissues (e.g., the vascular structures), where the SA-PAM mechanism could find its applications.

SA-PAM can also be refined in other dimensions. For instance, to obtain the height imaging and boundary detection results shown in Fig. 3, Fig. 4, we utilize a second scheme with three fixed transducers that simultaneously record signals $P_{l}$, $P_{r}$ and $P_{c}$, eliminating the need for transducer rotation. The 2D scanning for image acquisition is achieved using a motorized stage. To enable real-time SA-PAM, faster scanning mechanisms can be implemented [39], [44], [45], [46]. The full angular patterns shown in Figs. 2(b), 3(b) and 4(b) encode rich information. For future applications requiring continuous monitoring such far-field angular patterns, the system can be optimized by employing a transducer array to collect data from multiple angles simultaneously.

### 5.2 Other potentials of evanescent PA waves

In this work, we demonstrate the generation and detection of evanescent photoacoustic (PA) waves in photoacoustic microscopy (PAM), revealing their unique and intriguing role in the PA process. These evanescent PA waves are universally generated when the laser is tightly focused to a spot size smaller than the acoustic wavelength of interest. Furthermore, local structures with dimensions below the acoustic wavelength, such as microscopic features in biological samples, can serve as subwavelength PA sources, generating evanescent waves under wide-field light illumination.

These evanescent PA waves can be redirected into the far-field using adjacent local structures. As an example, we utilize an interface to demonstrate how evanescent PA waves can be redirected into the far-field beyond the critical angle. We also highlight that other approaches, such as local probes akin to those used in scanning optical near-field microscopy [47], [48], [49], can be employed to achieve similar effects. By integrating such probes into PAM, super-resolution imaging—beyond the diffraction limit imposed by the acoustic wavelength—can be realized by directly detecting evanescent PA waves, eliminating the need for precise laser focusing.

The implications of evanescent PA waves extend far beyond PAM, offering potential applications in a variety of advanced technologies. For instance, evanescent PA waves generated by structured light could be harnessed for precision manipulation in optical tweezer systems [50], [51]. In summary, the distinctive properties of evanescent PA waves pave the way for innovative imaging and manipulation techniques, broadening the scope of their applications in both scientific and technological domains.

## CRediT authorship contribution statement

**Xing Yiheng:** Resources. **Xiao Jingtao:** Software, Methodology. **Chen Chang:** Resources. **Xu Hongxing:** Supervision. **Shen Hao:** Supervision. **Shen YueCheng:** Supervision. **Li Beibei:** Software, Methodology, Formal analysis. **Zhang Liying:** Validation, Formal analysis, Data curation. **Zhou Rong:** Writing – original draft, Visualization, Project administration, Methodology, Data curation. **Pan Deng:** Writing – review & editing, Supervision, Funding acquisition, Conceptualization.

## Declaration of Competing Interest

The authors declare the following financial interests/personal relationships which may be considered as potential competing interests: Deng Pan reports financial support was provided by the Ministry of Science and Technology of China. If there are other authors, they declare that they have no known competing financial interests or personal relationships that could have appeared to influence the work reported in this paper.

## Data Availability

Data will be made available on request.
