# Supplementary material for: Harnessing evanescent photoacoustic waves for multi-domain imaging
Source: Photoacoustics. 2025 Mar 29;43:100719. doi: 10.1016/j.pacs.2025.100719 (PMC12004371; doi:10.1016/j.pacs.2025.100719)
Supplement: Supplementary file 1 — Supplementary material [file mmc1.docx]

**Supplementary notes for “Harnessing evanescent photoacoustic waves for multi-domain imaging”**

**Table of Contents**

[Supplementary Note 1: Layout of angle-resolved PAM 2](#_Toc193145369)

[Supplementary Note 2: Frequency spectrum of PA signals 3](#_Toc193145370)

[Supplementary Note 3: Simulation model and results analysis 4](#_Toc193145371)

[Supplementary Note 4: Fabrication of glass wedge 8](#_Toc193145372)

[Supplementary Note 5: Depth dependence in epoxy resin 9](#_Toc193145373)

# Supplementary Note 1: Layout of angle-resolved PAM


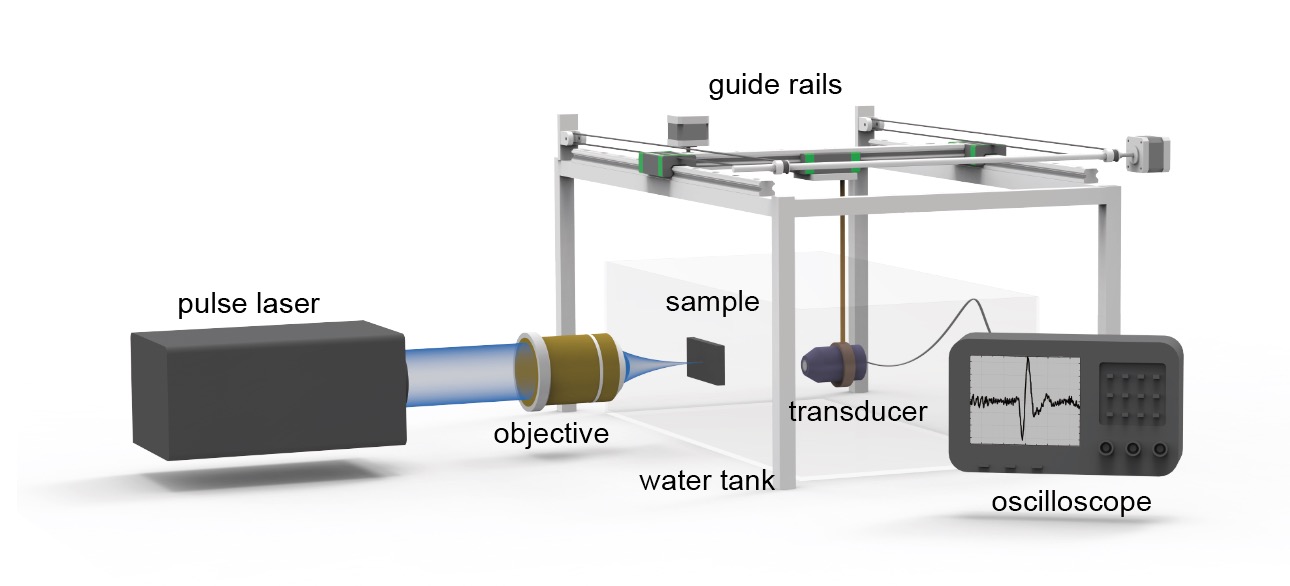


**Figure S1.** Schematics of the setup of angle-resolved PAM. Detailed descriptions of the setup are provided in Method section in the main text.

# Supplementary Note 2: Frequency spectrum of PA signals

The PA signals, generated by the pulsed laser, manifest as pulses of acoustic waves across a spectrum of frequencies. To analyze the spectrum of these signals, we conducted an experiment using a bare black tape, as illustrated in Fig. S2(a). A pulsed laser was directed onto the tape, generating the PA signal pulses. These pulses were captured temporally by a transducer connected to an oscilloscope, as shown in Fig. S1, oriented along the normal direction. The typical spectrum of the sample is presented in Fig. S2(b), with the recorded photoacoustic signal spectrum centered at 5 MHz and extending up to 9 MHz. We note that this spectrum is primarily influenced by the sensitivity of the transducer, with the chosen transducer exhibiting maximum sensitivity at 5 MHz.


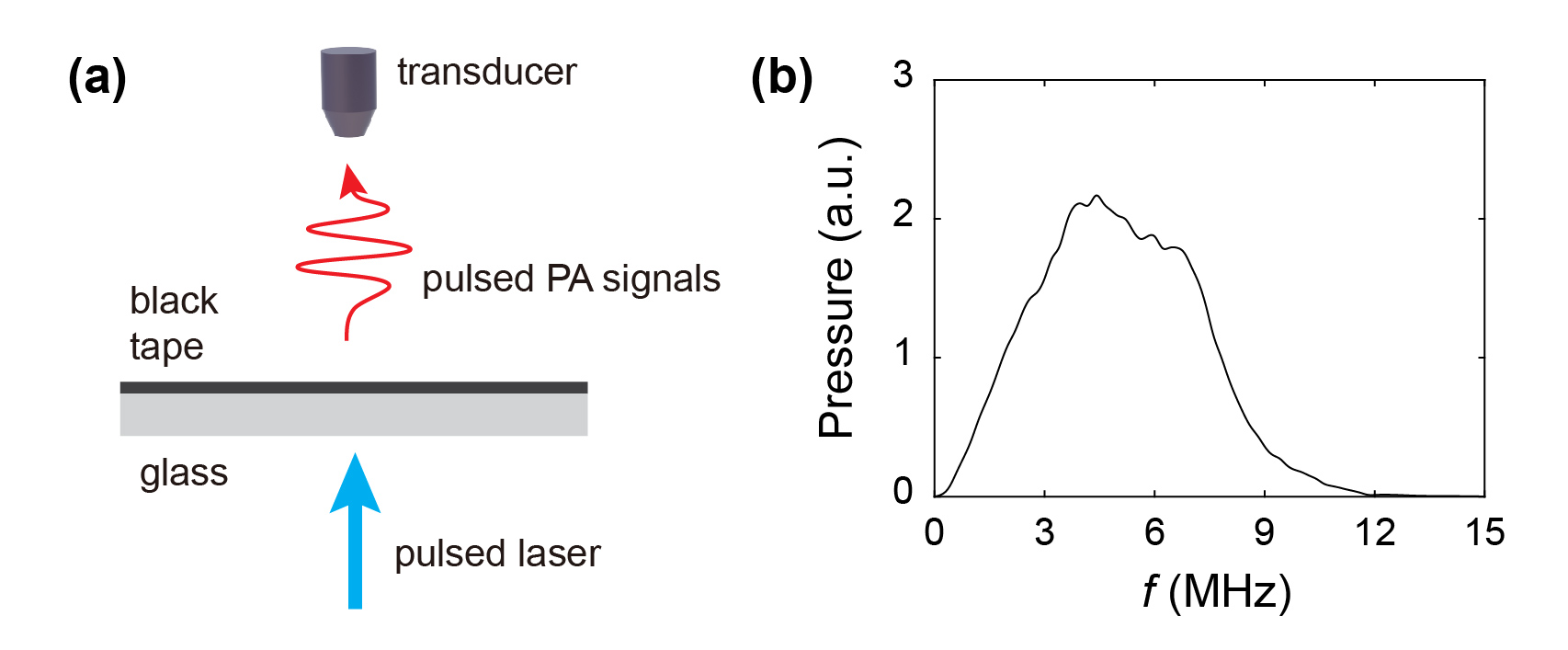


**Figure S2**. (a) Schematics of the experimental setup. **(b)** The frequency spectrum of the recorded PA signals.

# Supplementary Note 3: Simulation model and results analysis

**1. Modelling methods**

The numerical simulation results presented in Fig. 1(a) are obtained using the finite element method (COMSOL Multiphysics^TM^ 6.0, pressure acoustic module). In this approach, we model sound waves within the glass as pressure acoustics and those within the water as elastic waves, interconnected through an acoustic-solid interface. The acoustic point-like sources are located within the glass region and adjacent to the water region. A perfectly matched layer envelops the simulation domain to mitigate reflections. Following field calculation within this domain, the far-field angular distribution can be derived by evaluating the Helmholtz-Kirchhoff integral.

In the simulation, the mass densities are 1000 kg/m³ for water and 2200 kg/m³ for glass, respectively. The sound speed in water, denoted as $c_{w}$, is a consistent value of 1480 m/s across various literatures. In contrast, sound speeds in glass exhibit variability due to dependence on fabrication process parameters. For our simulation, we chose a S-wave speed of $c_{s}=2958$ m/s for glass, aligning with experimental observations, such as the observation angle of far-field minima and the exponential decaying length illustrated in Fig. 3(c). Similarly, we set the P-wave speed in glass to $c_{p}=5710$ m/s, which marginally influences the resultant data.

To accurately model the photoacoustic source, we initially analyze the emission properties of a photo-generated point source through experimental means. We perform an angle-resolved PAM measurement of the photoacoustic emission directly released into the water semi-space. Illustrated in Fig. S3(a), our sample comprises black tape on a glass substrate without a cover glass. The directional emission of the point source towards a semi-space, as depicted in Fig. S3b, resembles half of a dipole emission pattern. This dipole-like emission signifies the anisotropic nature of the sample's thermal expansion, as further illustrated in Fig. S3(c). Longitudinal expansion along the z-direction induces velocity in that direction, thereby generating longitudinal waves. In contrast, lateral expansion minimally contributes to far-field signals due to the absence of shearing stiffness between the sample and the surrounding medium. This argument also holds true for samples layered between two pieces of glass, as utilized in the main text.

To capture the experimental details of the photoacoustic source, we model it as a localized distribution of initial velocities perpendicular to the interface. Such distribution of initial velocities can give rise to a dipolar emission. This model aligns with the observed far-field pattern in the water region (Fig. S3(b)) and our prior understanding on thermal expansion properties (Fig. S3(c)). However, it's worth noting that the emission in our experimental setup does not precisely mimic a dipole. For instance, in the case of a free-standing black tape, PA signals observed on both sides should ideally be in-phase, whereas a true dipole emission would exhibit out-of-phase emissions on either side. Nevertheless, given the thickness of the substrate and its treatment as semi-infinite, we adopt the dipole model to describe emissions towards the water region, yielding satisfactory agreement with experimental measurements.


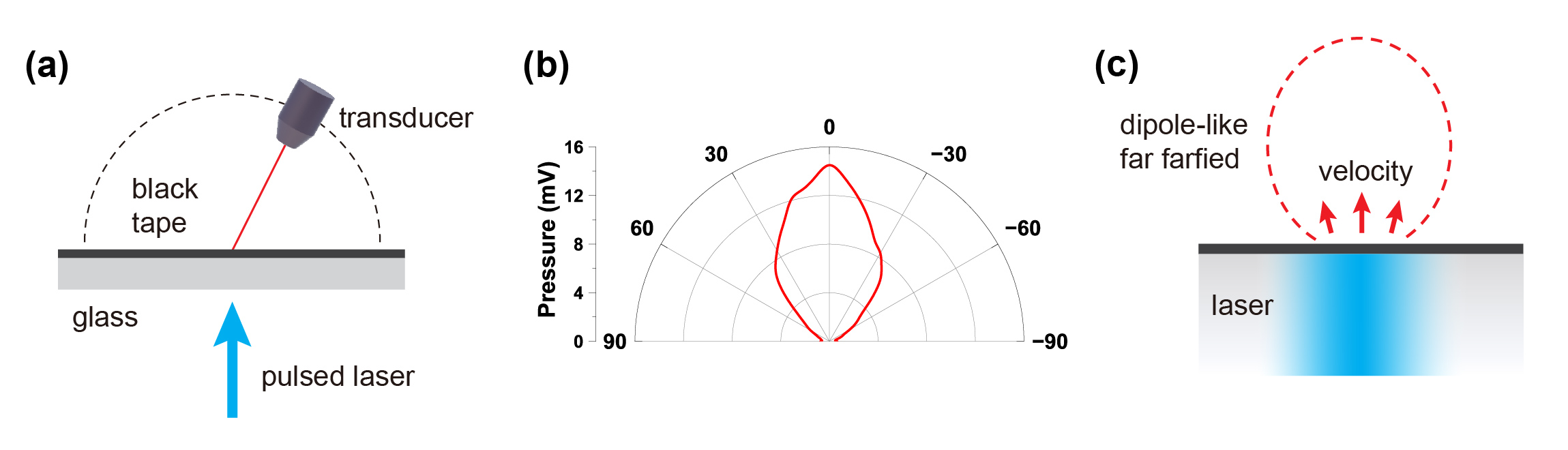


**Figure S3**. **(a)** Schematics of the experimental setup. **(b)** The angular distribution of the measured far-field PA signals. (c) Illustration of the thermal expansion caused by the laser, resulting in dipole-like far-field PA signals.

**2. Simulated field distributions**

The simulated field distributions, including both angular distributions in the far field and spatial distributions in the intermediate zone, for two typical source depths are presented in Fig. S4. The properties of far-field signals are primarily governed by the observation direction relative to critical angles. In solid materials like glass exhibiting both S- and P-waves, critical angles for both S- and P-waves can be determined by

$\theta_{\mathrm{cr}}^{S,P}=asin(c_{w}/c_{g}^{S,P})$,

where $c_{w}$ and $c_{g}^{S,P}$ denote the sound speeds in water (longitudinal wave) and glass (taking glass as an example), respectively. The velocities adhere to the relation $c_{g}^{P}>c_{g}^{S}>c_{w}$, resulting in $\theta_{\mathrm{cr}}^{S}>\theta_{\mathrm{cr}}^{P}$.


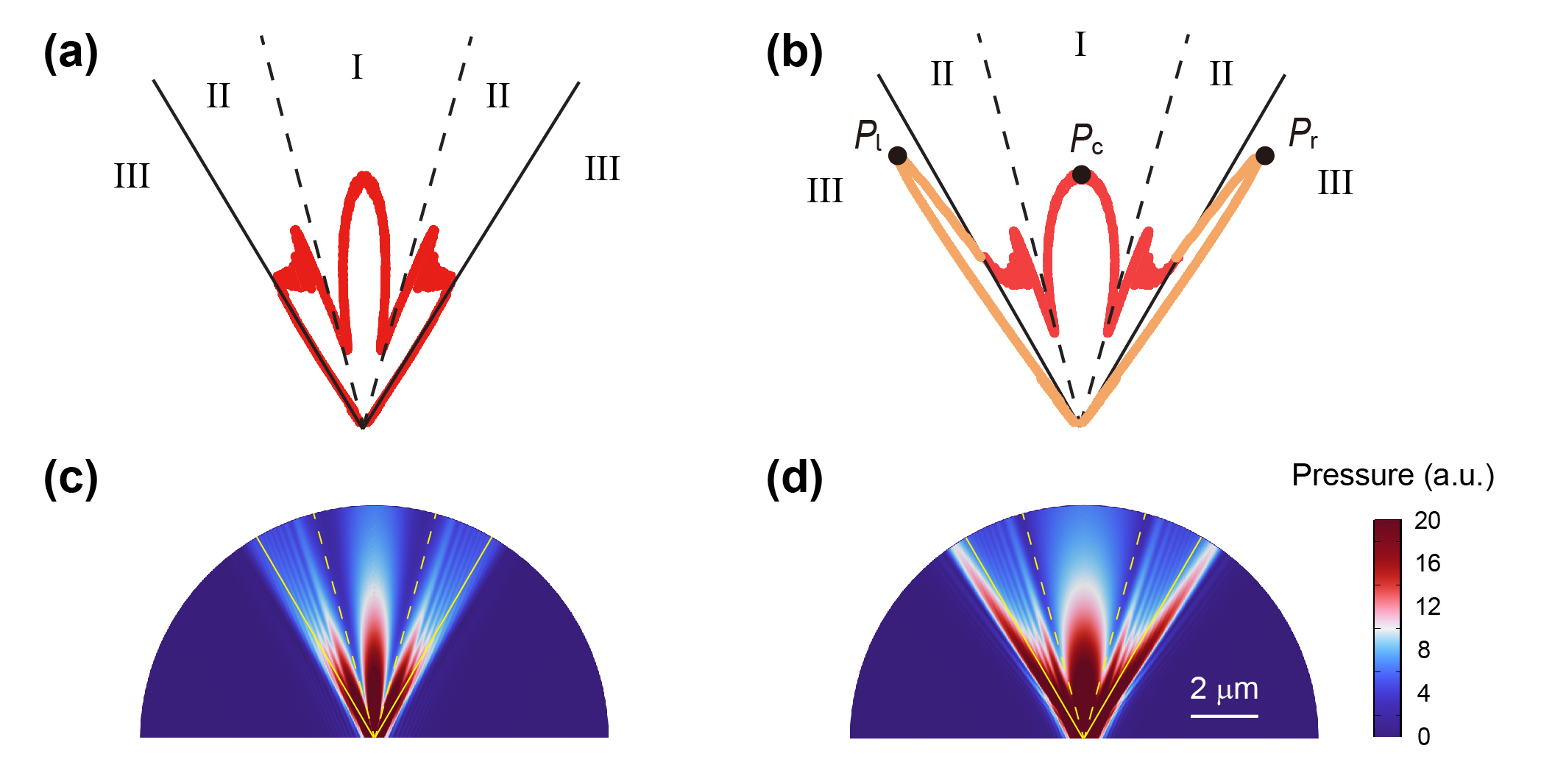


**Figure S4.** **(a, b)** Angular distributions of far fields for sources depths of *d*=600 μm (**a**) and *d*=200 μm (**b**) (adapted from Fig. 1**(a)**). **(c, d)** Spatial distributions for sources depths of *d*=600 μm (**c**) and *d*=200 μm (**d**).

In Fig. S4, the critical angles for the S- and P-waves are indicated by solid and dashed lines, respectively. According to the observation direction relative to these critical angles, as illustrated in Fig. S4 the far-field signals can be categorized into three regions:

1. Region I ($\theta<\theta_{\mathrm{cr}}^{P}$): the PA signals result from the superposition of under-critical angle transmissions of both propagating S- and P-waves.
2. Region II ($\theta_{\mathrm{cr}}^{P}<\theta<\theta_{\mathrm{cr}}^{S}$): the PA signals receive contributions from the under-critical transmission of the propagating S-wave, but the SA transmission of the evanescent P-wave.
3. Region III ($\theta>\theta_{\mathrm{cr}}^{S}$): the PA signals stem from the SA transmission of both the S- and P-wave.

As we have explained in the main text, minima are observed along the critical angles, in contrast to the case of an optical emitter near an interface. The SA signals above $\theta_{\mathrm{cr}}^{S}$ increases sharply and exhibit two peaks as the source approaches the interface. The dependence of emission on the source depth is further discussed in the following section.

# Supplementary Note 4: Fabrication of glass wedge

In Figure 3, we employed a wedge-shaped cover glass to introduce a continuous variation in the depths of the sample surface from the interface. This cover glass was prepared through wet etching, employing a 5-10% aqueous solution of hydrofluoric acid (CAS: 7664-39-3, Aladdin). To create the wedge glass, two glass slides of uniform thickness (700 μm) were bonded together and immersed vertically in the solution. Subsequently, the glass was gradually extracted from the solution at a constant rate for more than 3 hours using a step motor. This process resulted in a longer etching period for the lower section, yielding a wedge shape with variable thickness. The angle of the wedge could be adjusted by altering the pulling speed.


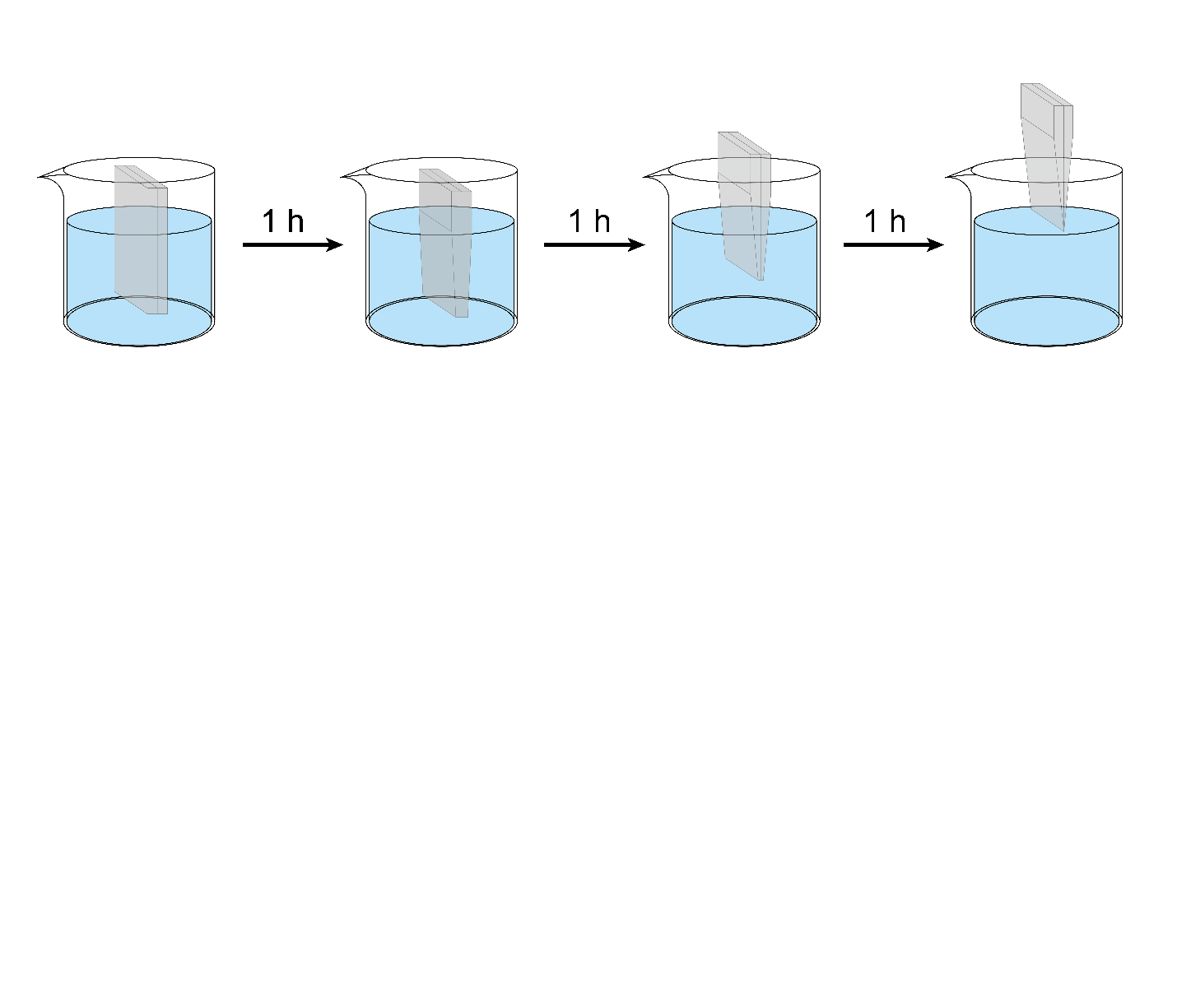


**Figure S6.** Illustration of the fabrication process for the glass wedge using the wet etching method.

# Supplementary Note 5: Depth dependence in epoxy resin

In Fig. 5, a sample of bee wing was embedded in epoxy resin material. To conduct depth measurements in SA-PAM, the dependence of the dimensionless factor $\eta$ on the source depths, as presented in Fig. 3 for glass, should be re-calibrated. We performed SA-PAM measurements for a bee wing sandwiched between an epoxy resin substrate and an epoxy resin cover plate of various thickness, with the measured values of *η* presented in Fig. S7. Consistent with the findings for glass material, the depth dependence of the factor *η* can be well-fitted by the exponential function, $\eta_{0}e^{-|\kappa|d}+b$, yielding $\eta_{0}=2.1583$, $\left| \kappa\right|=3.74$ mm^-1^, $b=0.2777$.


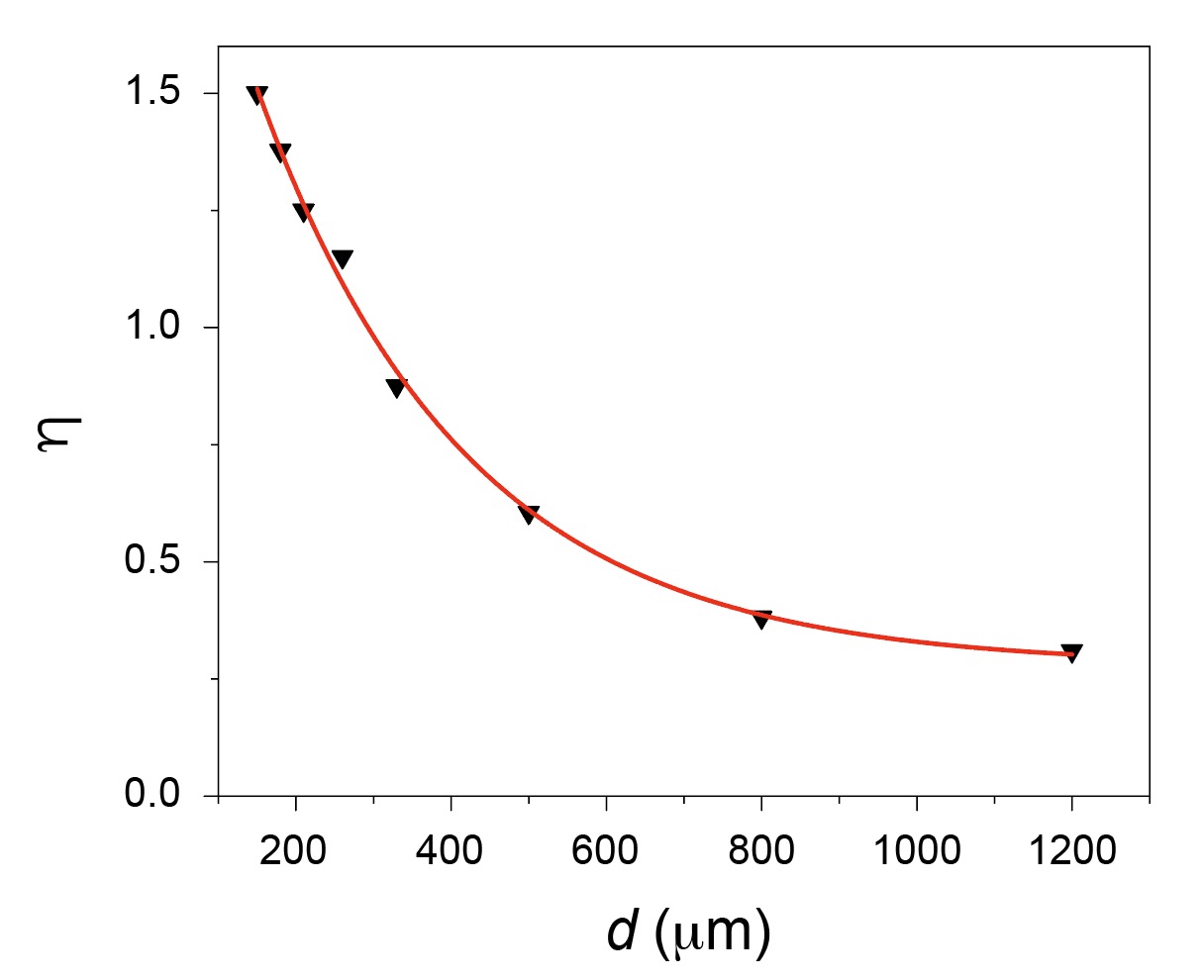


**Figure S7.** Experimental measurements of $\eta$ at varying source depths within an epoxy resin medium.
